# Combination of a Deep Eutectic Solvent and Macroporous Resin for Green Recovery of Iridoids, Chlorogenic Acid, and Flavonoids from *Eucommia ulmoides* Leaves

**DOI:** 10.3390/molecules29030737

**Published:** 2024-02-05

**Authors:** Yunhui Liao, Feng Chen, Haishan Tang, Wubliker Dessie, Zuodong Qin

**Affiliations:** 1 College of Chemistry and Bioengineering, Hunan University of Science and Engineering, Yongzhou 425199, China; liaoyunhui@huse.edu.cn (Y.L.); chenfeng1157354@163.com (F.C.); thshappy@163.com (H.T.); dwubliker@yahoo.com (W.D.); 2 Hunan Engineering Technology Research Center for Comprehensive Development and Utilization of Biomass Resources, College of Chemistry and Bioengineering, Hunan University of Science and Engineering, Yongzhou 425199, China; 3 Hunan Provincial Key Laboratory for Comprehensive Utilization of Dominant Plant Resources in Southern Hunan, Yongzhou 425199, China

**Keywords:** *Eucommia ulmoides* leaves (EULs), deep eutectic solvents, extraction, macroporous resin, biological activity

## Abstract

To increase the effectiveness of using typical biomass waste as a resource, iridoids, chlorogenic acid, and flavonoids from the waste biomass of *Eucommia ulmoides* leaves (EULs) were extracted by deep eutectic solvents (DESs) in conjunction with macroporous resin. To optimize the extract conditions, the experiment of response surface was employed with the single-factor of DES composition molar ratio, liquid–solid ratio, water percentage, extraction temperature, and extraction time. The findings demonstrated that the theoretical simulated extraction yield of chlorogenic acid (CGA), geniposidic acid (GPA), aucubin (AU), geniposide (GP), rutin (RU), and isoquercetin (IQU) were 42.8, 137.2, 156.7, 5.4, 13.5, and 12.8 mg/g, respectively, under optimal conditions (hydrogen bond donor–hydrogen bond acceptor molar ratio of 1.96, liquid–solid ratio of 28.89 mL/g, water percentage of 38.44%, temperature of 317.36 K, and time of 55.59 min). Then, 12 resins were evaluated for their adsorption and desorption capabilities for the target components, and the HPD950 resin was found to operate at its optimum. Additionally, the HPD950 resin demonstrated significant sustainability and considerable potential in the recyclability test. Finally, the hypoglycemic in vitro, hypolipidemic in vitro, immunomodulatory, and anti-inflammatory effects of EUL extract were evaluated, and the correlation analysis of six active components with biological activity and physicochemical characteristics of DESs by heatmap were discussed. The findings of this study can offer a theoretical foundation for the extraction of valuable components by DESs from waste biomass, as well as specific utility benefits for the creation and development of natural products.

## 1. Introduction

In China, *Eucommia ulmoides* (*E. ulmoides*) has been employed as a traditional Chinese medicine for more than 2000 years [1,2,3]. It has a variety of biological activities including antioxidative [4,5], anti-hypertensive [6,7], anti-inflammatory [8,9], and anti-obesity [10,11] impacts to fortify the immune system and internal organs of humans. When considering the availability of resources, *E. ulmoides* leaves (EULs) have been regarded as a very useful option compared to *E. ulmoides* bark due to their rich resources and excellent ability to regenerate and recover [12]. Thus, choosing an efficient extraction agent for EUL is currently the focus of research.

Because of their low toxicity and excellent biodegradability, deep eutectic solvents (DESs) are gaining popularity in comparison to traditional organic solvents, particularly for the extraction of natural products [13,14]. DESs are a eutectic mixture composed of two or more hydrogen-bonded components [15,16]. They have sparked great interest since they are liquid at normal temperature comparable to other solvents. The hydrogen bond acceptor (HBA) and hydrogen bond donor (HBD) are DES components that can be biodegraded and satisfy the standards of green chemistry due to providing natural sources of ingredients, such as in animal feed additives (choline chloride, ChCl), fertilizers (urea), sweeteners (glycerol), antifreeze (ethylene glycol), and plant metabolites (sugars, glycols, and organic acids) [17]. Among them, choline chloride and urea were the most common DESs used for extraction as HBA and HBD, respectively [18,19]. Additionally, DESs are predicted to replace conventional solvents with a greener alternative due to their excellent designability and straightforward preparation method [20], despite some emerging eutrophication problems [21]. The strong hydrogen bonds between two or more components of DESs can enhance their penetration capacity and accelerate the dissolution of active ingredients [22,23].

Iridoids, chlorogenic acid, and flavonoids are the main phytochemicals in EULs [24,25]. However, the conventional isolation of bioactive compounds from EUL extract using activity-guided fractionation and isolation is a time-consuming, laborious, and expensive process, and their activity could be lost due to dilution effects or decomposition during the isolation and purification, particularly for antioxidants. Therefore, simple, fast, and efficient ways to separate and purify potential active components from complicated extracts using macroporous resin are crucial to avoid the aforementioned issues [26,27].

Currently, few investigations have been completed on the extraction and purification of EUL extract using macroporous resin and DESs [28], while most studies have reported ethanol extraction [29,30], choline tryptophan ionic liquid (IL) extraction [31], ionic liquid-based enzyme-assisted extraction [32], microwave ultrasonic solvent extraction [28], and mesoporous carbon enrichment [33]. Moreover, there are also issues with the use and recycling of macroporous resin [34,35]. Therefore, this work aims to investigate an effective method for enriching and separating iridoids, chlorogenic acid, and flavonoids from EULs based on macroporous resin. For this purpose, we examined and compared the adsorption and desorption of 12 different kinds of macroporous resin. Following that, the recyclability of resins was also taken into consideration. Finally, the hypoglycemic in vitro, hypolipidemic in vitro, immunomodulatory, and anti-inflammatory effects of EUL extract were further evaluated, which established and developed an effective strategy for the application of natural products.

## 2. Results and Discussion

### 2.1. Selection of DESs

Since DESs have distinct physicochemical characteristics in terms of pH, polarity, and viscosity, their structure is critical to extraction efficiency [36]. This study aimed to evaluate the effectiveness of 10 DESs in extracting iridoids, chlorogenic acid, and flavonoids from EULs. The high viscosity of DESs has been noted as restricting their usage due to mass transfer obstruction [37,38,39]. Therefore, a 20% (*v*/*v*) moisture content in combination with several DESs was employed. Moreover, it is critical to consider the thermal stability and eutectic point of DESs when assessing their suitability as a solvent [40]. Figure S1 shows the thermogravimetric analysis (TGA) curves of the DESs. In the temperature range of 353.15 to 393.15 K, the weight loss of both DESs were generally attributed to the evaporation of free water. The first breakdown of DESs with weight loss was between 393.15 and 573.15 K. As a result, DESs may maintain their structure and functionality during the separation process because they are thermally stable. In addition, Figure S2 displays the DSC thermograms; it can be seen that the melting point (T_m_) of DESs is significantly lower than the melting point of any constituents and consistent with theoretical predictions of a drop in melting point [41].

**Figure 1:**
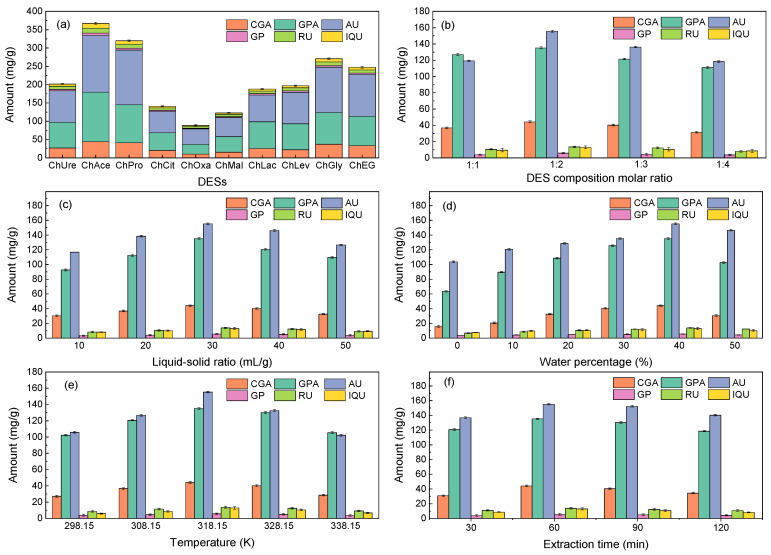
The effect of different DESs on iridoids, chlorogenic acid, and flavonoids yield (**a**) and single-factor analysis of the extraction procedure (**b**–**f**). Extraction conditions: (**a**) the HBA-HBD molar ratio was 1:2, the liquid–solid ratio was 30 mL/g, the water percentage was 40%, the temperature was 318.15 K, and the time was 60 min; (**b**) the liquid–solid ratio was 30 mL/g, the water percentage was 40%, the temperature was 318.15 K, and the time was 60 min; (**c**) the HBA-HBD molar ratio was 1:2, the water percentage was 40%, the temperature was 318.15 K, and the time was 60 min; (**d**) the HBA-HBD molar ratio was 1:2, the liquid–solid ratio was 30 mL/g, the temperature was 318.15 K, and the time was 60 min; (**e**) the HBA-HBD molar ratio was 1:2, the liquid–solid ratio was 30 mL/g, the water percentage was 40%, and the time was 60 min; (**f**) the HBA-HBD molar ratio was 1:2, the liquid–solid ratio was 30 mL/g, the water percentage was 40%, and the temperature was 318.15 K.

The extraction efficiency of different DESs are depicted in Figure 1a and the physicochemical properties are listed in Table 1. The findings showed that ChAce had the highest extraction yield for iridoids’, chlorogenic acid, and flavonoids’ extraction. The pH of DESs could affect the way solutes and solvents interact intermolecularly, resulting in varying levels of target chemical extraction efficiency from the sample matrix [42]. Therefore, with a pH of around 3, ChAce exhibited better extraction efficiency than other DESs, as demonstrated in Table 1. In addition, it was found that the increase in the amount of iridoids, chlorogenic acid, and flavonoids after extraction was in line with the increased specific surface area (S_BET_) and pore volume (V_pore_) of EULs. This could be because the structure of the cell wall was destroyed, making it easier for the DESs to permeate through the cells, lowering the barrier of mass transfer and accelerating the leaching of iridoids, chlorogenic acid, and flavonoids.

**Figure 2:**
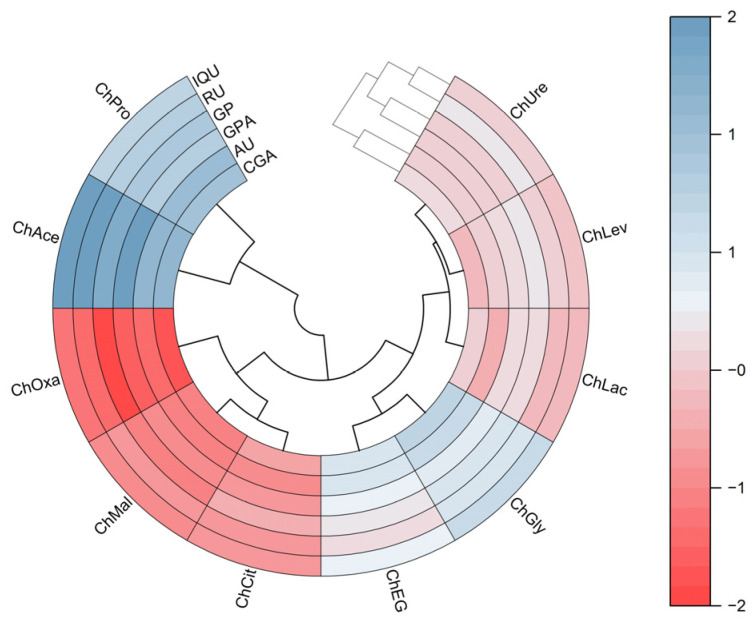
Heatmap analysis for different DESs on the yield of iridoids, chlorogenic acid, and flavonoids.

To illustrate the results, the yields of active components were standardized and shown as a heatmap (Figure 2). The DES samples are divided into three clusters. The ChPro and ChAce samples are isolated from all other clusters and have a high positive correlation with each other. Then, the ChOxa, ChMal, and ChCit can be grouped in the same cluster. Additionally, it is likely to group the ChEG, ChGly, ChLac, ChLev, and ChUre in the same cluster based on similarities. Compared to all other DESs, it was found that the ChAce was favorably correlated with the yield of iridoids, chlorogenic acid, and flavonoids, thus exhibiting significant differences in extraction efficiency.

### 2.2. Investigation of the Extraction Process by Single-Factor

The surface tension and viscosity of solvents can be impacted by variations in the molar ratio of choline chloride to hydrogen bond donors [43,44,45]. Thus, the impact of the DES composition molar ratio on the extraction yield was examined. Figure 1b illustrates that the optimal molar ratio of HBA-HBD was 1:2. Moreover, the impact of HBA-HBD molar ratio on thermal stability and eutectic point of DESs is depicted in Figures S3 and S4; the results show that all prepared DESs have good thermodynamic stability and exerted the lower melting point than their pure component.

Figure 1c illustrates the trend of extraction efficiency of six target components, which increased with the liquid–solid ratio before stabilizing, then an inflection point occurred at 30 mL/g. The extraction of six target components was facilitated by an appropriate ratio of liquid to solid. In this work, the active components were completely extracted at 30 mL/g. Therefore, the optimal liquid–solid ratio was 30 mL/g.

The high viscosity of DESs has been noted to restrict their usage due to mass transfer obstruction [37,38,39]. Thus, the impact of water percentage on extraction efficiency is demonstrated in Figure 1d. The results show that as the proportion of water increased, the extraction yield increased as well, reaching its maximum value at 40%. Usually, there was a transition from the water-in-DES behavior to the DES-in-water behavior with increased water content [46,47]. In the range of 0–40% water percentage, the increased in-water content could efficiently alter the polarity and viscosity of DESs. The hydrogen bonding between DESs and active components was enhanced, and thus increased the extraction yield [48]. However, when the water percentage increased to 50%, the DESs may be disrupted as the water-in-DES behavior predominated, resulting in an aqueous solution of DESs. Therefore, the interactions between DESs and target components may be weakened and broken by an excessive amount of water, leading to a reduced extraction efficiency [38,49].

For efficient mass transfer, the extraction temperature is crucial. The extraction yield rose to 318.15 K and subsequently reduced to 328.15 K (Figure 1e). This phenomenon can be explained by rising temperatures leading to the increase in thermal impact, which promoted the leaching of target components and increased the extraction yield. Meanwhile, the reduced extraction yield of the target components resulted from the degradation of some target components and increased the dissolution of impurities as the temperature further increased.

Figure 1f shows the impact of extraction time on extraction efficiency. The extraction yield initially increased and subsequently decreased. It is possible that the longer extraction time enhanced the extraction efficiency. However, after the extraction time exceeded 60 min, it might cause the decomposition of certain components and the exudation of undesirable components [31]. As a result, 60 min was determined to be the ideal extraction time.

### 2.3. Response Surface Methodology (RSM)-Based Extraction Condition Optimization

#### 2.3.1. Quadratic Multiple Regression Model Analysis

After fitting regression, the results of the ANOVA analysis were summarized in relation to each factor (Supplementary Table S1). The *p*-value of six target chemical extraction models were less than 0.0001, suggesting a high degree of dependability in the regression equation. The actual value is extremely consistent with the anticipated value, as indicated by the lack of fit item *p* > 0.05, so the model has a high degree of fitting.

#### 2.3.2. Determination and Verification of Optimized Conditions

The model equations for six target components are listed in Table S2 and the response surface map is depicted in Figures S5–S10. Using a 3D surface diagram and independent variable regression analysis, the optimal parameters were found. Under the optimized conditions (HBD-HBA ratio of 1.96, liquid–solid ratio of 28.89 mL/g, water percentage of 38.44%, temperature of 317.36 K, and time of 55.59 min), the theoretical simulated yield of CGA, GPA, AU, GP, RU, and IQU were 42.8, 137.2, 156.7, 5.4, 13.5, and 12.8 mg/g, respectively. The verification test was conducted to evaluate the reliability of the data produced by RSM, and the results are summarized in Table S3. Under the actual verification conditions (HBD-HBA ratio of 1.96, liquid–solid ratio of 28.89 mL/g, water percentage of 38.44%, temperature of 317.15 K, and time of 55.60 min), the average yield of CGA, GPA, AU, GP, RU, and IQU were 42.4, 136.7, 154.9, 5.2, 13.2, and 12.6 mg/g, respectively. Furthermore, the significance of the prediction model is certified by the relative standard deviation (RSD%) of 0.73%. Moreover, we compared our work with extraction data from the previous literature and found that our extraction yield was 10 times greater than that published in the literature [12,32,50].

### 2.4. Adsorption Capacity and Desorption Ratio

**Figure 3:**
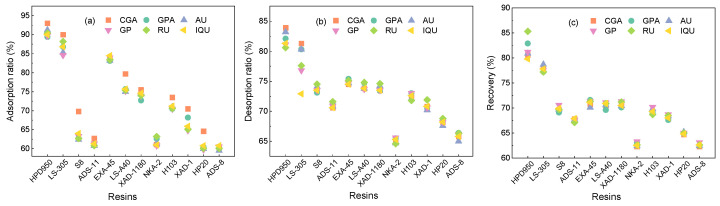
Adsorption (**a**) desorption (**b**), and recovery (**c**) of target components in resins.

**Figure 4:**
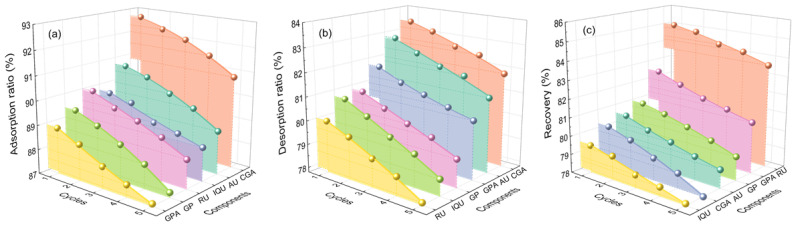
Recyclability results of HPD950 resins on adsorption (**a**), desorption (**b**), and recovery (**c**).

Figure 3a shows the absorption capacity of six target components in different macroporous resins. It was discovered that the polar resins had a higher absorption capacity than the other resins due to their bigger surface area and pore size according to Table 2. The process of adsorption was enhanced by resins with similar polarity and greater average pore diameters and surface areas [51]. Also, investigations into the desorption capacity are depicted in Figure 3b. The desorption ratio of active components was directly proportionate to the pore size of resins, among which the HPD950 resin exerted the best desorption performance. This can result from the physical force (such as the van der Waals force) and the influence of phenolic hydroxyl groups. Therefore, the HPD950 resin was most effective in recovering target compounds, and the recovery yields of 80.8%, 82.9%, 81.2%, 80.6%, 85.3%, and 79.8% were obtained for AU, GPA, GP, CGA, RU, and IQU (Figure 3c), respectively. Meanwhile, the fifth cycle in Figure 4 demonstrated extraordinary stability and repeatability. Therefore, the HPD950 resin had great sustainability and showed considerable promise for use in the recovery of iridoids, flavonoids, and chlorogenic acid.

### 2.5. In Vitro Hypoglycemic and Hypolipidemic Effect

#### Inhibition of α-Glucosidase and α-Amylase Activity

**Figure 5:**
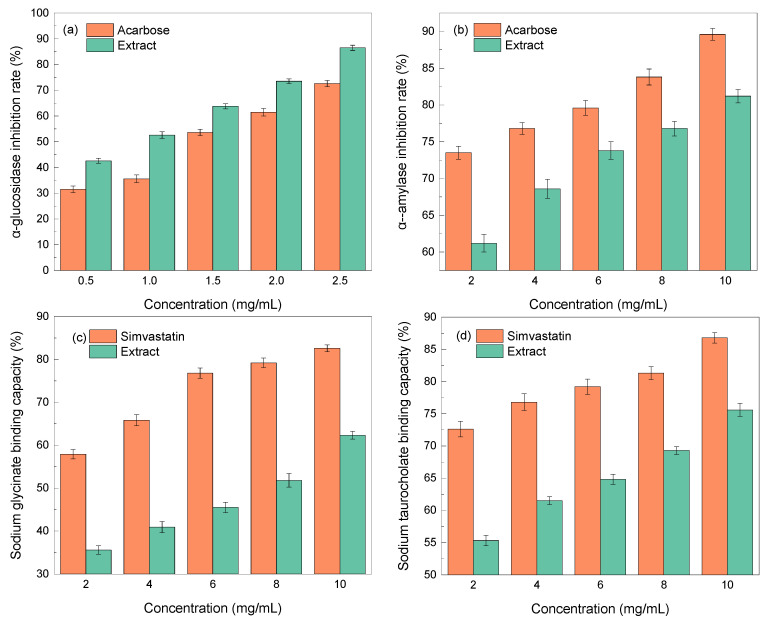
In vitro hypoglycemic and lipid-lowering activity of EUL extract: (**a**) α-glucosidase inhibition; (**b**) α-amylase inhibition; (**c**) sodium glycinate binding capacity; and (**d**) sodium taurocholate binding capacity.

The two main enzymes involved in the body’s metabolism of glucose are α-glucosidase and α-amylase. They can accurately lower blood glucose levels by limiting enzyme activity, lowering glucose absorption, as well as delaying the transformation of glucose into blood glucose [52]. Figure 5a,b depict the inhibitory impacts of EUL extract on α-glucosidase and α-amylase. It can be seen that the EUL extract had a substantial concentration-dependent inhibitory impact on α-glucosidase and α-amylase, which was a significant improvement over the positive control, acarbose.

The effectiveness of medicine in relation to hypolipidemic activity has been frequently assessed using the cholate binding technique. According to the theory, the concentration of bile salts in the body could decrease once the medication was combined with bile salts, in order to sustain the bile acid balance in the body, encourage the fat-breaking in the liver, and ultimately help lower blood lipid levels [53]. Sodium glycocholate and sodium taurocholate comprise the majority of bile salts, which play a significant role in human bile production and lipolysis. With an increase in concentration, the ability of the EUL extract to adsorb taurocholate and glycocholate increased, demonstrating a substantial dose–effect relationship, as shown in Figure 5c,d. Overall, EUL extract exhibited notable hypolipidemic effects in vitro.

### 2.6. Immunomodulatory Activity and Anti-Inflammatory Test

**Figure 6:**
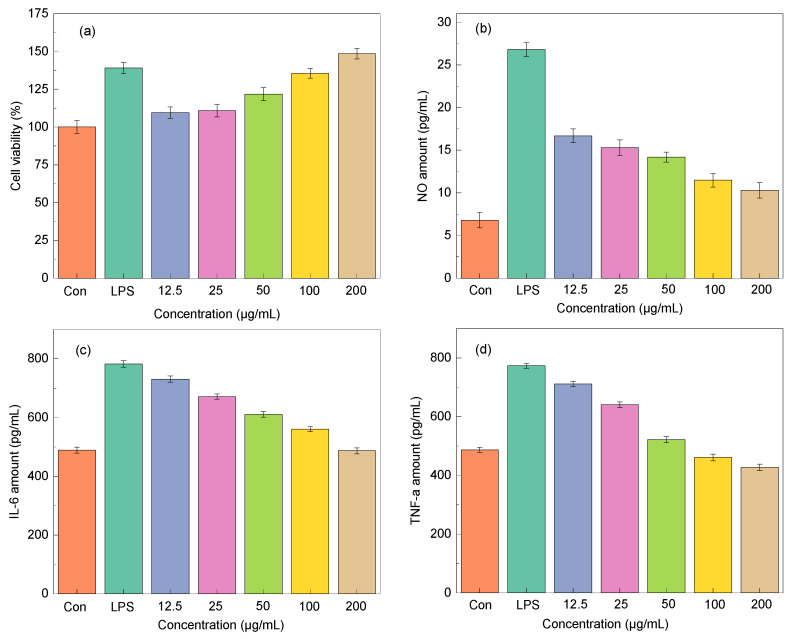
The impact of EUL extract on cell viability (**a**) and anti-inflammatory (**b**–**d**).

Macrophages are essential to the innate immune system. The majority of immunological functions, including host inflammation, are controlled by these cells. Therefore, the extract was used to examine the effects on cell activity and proliferation of RAW264.7 cell. It was found that the EUL extract boosted RAW264.7 cell proliferation within a certain concentration range, as shown in Figure 6a. At the concentration of 200 μg/mL, the EUL extract greatly increased RAW264.7 cell proliferation to the same extent as LPS in comparison to uninfected controls.

Aiming to test the anti-inflammatory activity of EUL extract, the inhibitory capability of NO, IL-6, and TNF-α in LPS-stimulated RAW264.7 cells was evaluated. As depicted in Figure 6b–d, LPS stimulation substantially enhanced the production of NO, IL-6, and TNF-α in macrophages. Then, the release of NO, IL-6, and TNF-α in macrophages was considerably reduced following the EUL extract culture, which suggested that the EUL extract has an inhibitory impact on macrophage inflammatory factors.

### 2.7. Correlation Analysis

**Figure 7:**
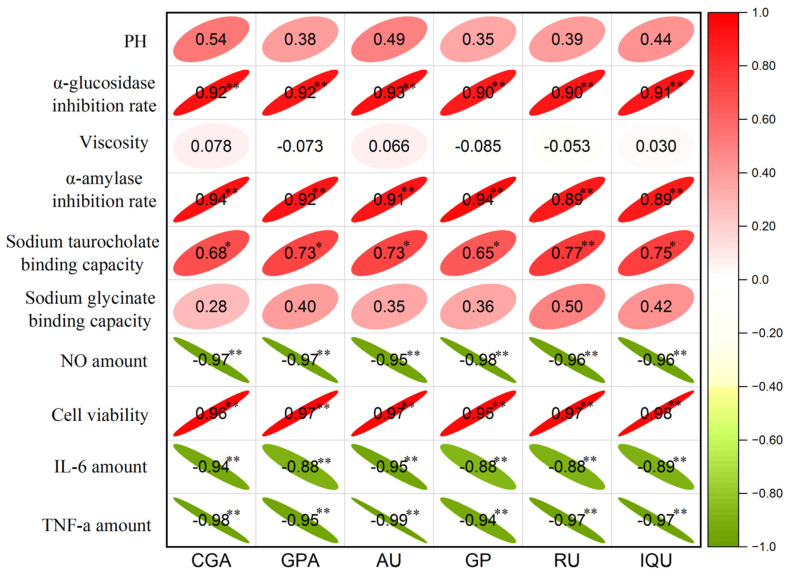
The correlation matrix of six active components with the biological activity of EUL extract and the physicochemical characteristics of DESs by a heatmap. The significant correlation at *p* < 0.05 and *p* < 0.01 levels is indicated by the * and **, respectively.

In order to examine the link between six active components with the biological activity of EUL extract and the physicochemical characteristics of DESs, the correlation coefficients of Pearson were obtained and the results are displayed as a heatmap (Figure 7). A strong association between active components and biological activity was found by correlation analysis. The α-glucosidase activity, α-amylase activity, and cell viability were positively correlated with all six active components (*p* < 0.01). The sodium taurocholate binding capacity was positively associated with RU (*p* < 0.01), CGA (*p* < 0.05), GPA (*p* < 0.05), AU (*p* < 0.05), GP (*p* < 0.05), and IQU (*p* < 0.05). While the NO amount, IL-6 amount, and TNF-α amount was negatively associated with all six active components (*p* < 0.01).

## 3. Material and Methods

### 3.1. Raw Materials and Chemicals

The EUL raw material was acquired from Zhangjiajie (Hunan, China). Before use, the EUL raw material was dried at 333.15 K for 12 h in a vacuum drying oven, ground, sieved through a 200–300 μm mesh, and then kept in a vacuum drier. The macroporous resins were purchased from Beijing Solebo Technology (Beijing, China). Choline chloride, urea, acetic acid, oxalic acid, propionic acid, lactic acid, citric acid, malic acid, levulinic acid, glycerol, ethylene glycol, simvastatin, glucosidase, amylase, sodium taurocholate, and sodium glycocholate were acquired from Aladdin Bio-Chem Technology Co., Ltd. (Shanghai, China). Chlorogenic acid (CGA), geniposidic acid (GPA), aucubin (AU), geniposide (GP), rutin (RU), isoquercetin (IQU), and ascorbic acid (Vc) standards were purchased from the China National Pharmaceutical Group Chemical Reagent Co., Ltd. (Shanghai, China), and other reagents were utilized without additional purification after being bought from Fuchen Chemical Reagent Co., Ltd. (Tianjin, China). Throughout the whole experimental process, fresh deionized water was utilized.

### 3.2. Preparation and Characterization of Deep Eutectic Solvents

The HBA (choline chloride, ChCl) and different types of HBD (urea, acetic acid, propionic acid, citric acid, oxalic acid, malic acid, lactic acid, levulinic acid, glycerol, and ethylene glycol) were added and stirred with a magnetic stirrer at 353.15 K for 30 to 60 min to generate a clear liquid [43,54]. After that, 20% (*v*/*v*) of water was added and stirred for 30 min to form homogeneous and clear DESs (ChUre, ChAce, ChPro, ChCit, ChOxa, ChMal, ChLac, ChLev, ChGly, and ChEG). The viscosity of DESs were measured by a viscometer (NDJ-5S, China). Thermogravimetric analysis (TG) curves were performed in an N_2_ atmosphere using a thermal analyzer (Mettler TGA-2; Switzerland) from room temperature to 873.15 K. Additionally, differential scanning calorimetry (DSC) curves were obtained by a DSC instrument (Netzsch STA 449C; Selb, Germany).

### 3.3. Preparation of Standard Solution

The standard was accurately weighed and sonicated with methanol. Subsequently, it was filtered by 0.22 μm microporous membrane (Jinteng Co. Ltd., Tianjin, China) and employed as the standard solution [12,55].

### 3.4. Characterization of EUL after Extraction

The Brunauer–Emmett–Teller (BET) surface areas and pore volumes of samples were determined by the Micromeritics TriStar II 3020 analyzer. Before determination, the samples were subjected to degassing for 12 h at 373.15 K.

### 3.5. Selection of Optimal DESs

A total of 5 g of EULs was added to a flask, then DESs were added under the identical factors (1:2 for the HBA-HBD molar ratio, 30 mL/g for the liquid–solid ratio, 40% for the water percentage, 318.15 K for the extract temperature, and 60 min for the extraction time). Subsequently, the extract was filtered, and the residue was washed with water before being dried to a constant weight. After filtering through the 0.22 µm microporous membrane, the extracted solution was ultimately tested by high-performance liquid chromatography (HPLC).

### 3.6. Single-Factor Experiment Design

A total of 10 g of EUL extract was added to a flask. Next, the DESs were added. The effects of the following factors on the extraction efficiency were evaluated: liquid–solid ratio (10, 20, 30, 40, and 50 mL/g), water percentage (0, 10, 20, 30, 40, and 50%), extraction temperature (298.15, 308.15, 318.15, 328.15, and 338.15 K), and time (30, 60, 90, and 120 min). In particular, 20% of water is always added apart in research on the effect of water. All data were recorded three times in each experiment.

### 3.7. HPLC Analysis and Quantification

Before analysis, the standard and extract solutions were filtered by 0.22 μm microporous membrane. A Shimadzu LC-20A chromatographic system, equipped with an Amethyst C18-H (4.6 mm × 250 mm, 5 μm) series HPLC column (Sepax Technologies, Newark, NJ, USA), a DGU-20A 3 degasser, a SIL-20A auto sampler, an SPD-20A UV detector, and a Shimadzu LC-20AB binary pump, was used for the HPLC quantification. A gradient elution with the mobile phase of 0.5% phosphoric acid (A) and methanol (B) solution was conducted for the run time of 60 min. The specific parameters were as follows [12]: 10 μL for the injection volume; 1.0 mL/min for the flow rate; 0–18 min, 6–20% B; 18–30 min, 20–28% B; 30–40 min, 28–30% B; 40–55 min, 30–55% B; 55–60 min, 55–60% B; 60–65 min, 60–6% B; 298.15 K for the column temperature; 206 nm (AU), 238 nm (GPA and GP), 254 nm (RU and IQU), and 320 nm (CGA) for the detection wavelength. The HPLC chromatograms of standard compounds and EUL extract are displayed in Figure S11.

### 3.8. The Design of Response Surface Optimization

Based on the previous results of single-factor experiment, ChAce was selected as the optimal DES for EUL extraction. The specific design is shown in the Supplementary Materials.

### 3.9. Static Adsorption and Desorption Tests

A total of 30 mL of EUL extract (4.08 mg/mL) and 1 g of resin were mixed in a conical flask. Then, the flask was vibrated by a thermostatic water bath shaker for 24 h. The vibrating speed and temperature were 120 rpm and 298.15 K, respectively. After that, the resin was carefully cleaned with distilled water, and desorbed in 30 mL 95% ethanol solution for 24 h at 298.15 K with a stirring speed at 120 rpm.

### 3.10. Recyclability Tests

The resin was continuously loaded and recycled under the best procedure for the adsorption and desorption of EUL extract.

### 3.11. Hypoglycemic In Vitro

#### 3.11.1. Inhibition of α-Glucosidase Activity

A 96-well plate was filled with a total of 100 μL of potassium phosphate buffer (pH 6.8), 20 μL of 0.2 U/mL α-glucosidase (produced with potassium phosphate buffer), and 10 μL of 1.0 mg/mL reduced glutathione [40]. Then, 20 μL of various concentrations of EUL extract (0.5–2.5 mg/mL) were added, mixed, and incubated at 310.15 K for 15 min. Next, 20 μL of 2.5 mmol/L *p*-nitrophenyl-α-D-glucopyranoside (PNPG) and 80 μL of 0.2 mol/L Na_2_CO_3_ solution were added in sequence, the absorbance value was determined by the visible spectrophotometer at 405 nm. The inhibition rate was determined employing acarbose as the reference [52]:

α-glucosidase activity inhibition rate (%) = [1 − (A_i_ − A_i0_)/(A_j_ − A_0_)] × 100% (1)

where A_j_ represents the absorbance of enzyme, buffer, and substrate; A_0_ represents the absorbance of enzyme and buffer; A_i_ represents the absorbance of enzyme, sample, buffer, and substrate; and A_i0_ represents the absorbance of enzyme, sample, and buffer.

#### 3.11.2. Inhibition of α-Amylase Activity

A total of 100 μL of 0.2 U/mL α-amylase solution (pH 6.8) was made with phosphate buffer solution, and pipetted into a centrifuge tube. Then, 500 μL of various concentrations of EUL extract (2.0–10.0 mg/mL) were added, mixed, and maintained for 20 min at 310.15 K. Next, 100 μL of 10 g/L starch solution was added and stirred at 310.15 K for 10 min. Following that, 375 μL of 3,5-dinitrosalicylic acid (DNS) reagent was added, stirred, and allowed to react for 5 min in a bath of boiling water, then cooled by flowing water and centrifuged for 5 min. Finally, the value of optical density (OD) was determined at 540 nm. The inhibition rate was obtained using acarbose as the control:

α-amylase activity inhibition rate (%) = [1 − (A_i_ − A_i0_)/(A_j_ − A_0_)] × 100% (2)

where A_i_ represents the absorbance of sample, enzyme, starch, and DNS; A_i0_ represents the absorbance of sample, enzyme, and DNS; A_j_ represents the absorbance of enzyme, starch, and DNS; A_0_ represents the absorbance of enzyme and DNS.

### 3.12. Hypolipidemic In Vitro

To replicate the gastric digesting process, 0.5 mL of EUL extract (2.0–10.0 mg/mL), and 1.5 mL of artificial gastric fluid were pipetted into a centrifuge tube, and oscillated at 310.15 K for 1 h. After that, the pH of the mixture was adjusted to 6.3 using the 0.1 mol/L NaOH solution. To simulate the intestinal environment, 2.0 mL of artificial intestinal fluid was supplied for digestion at 310.15 K for 1 h. Following that, 2.0 mL of 0.3 mmol/L sodium glycocholate and sodium taurocholate were added to each sample, and then oscillated at 310.15 K for 1 h. The supernatant was utilized to determine the amounts of sodium glycocholate and sodium taurocholate by colorimetry after centrifugation. The calculation used simvastatin as the reference:

Cholate binding rate (%) = (A_i_ − A_0_)/A_i_ × 100% (3)

where A_i_ represents the absorbance of cholate addition; A_0_ represents the absorbance of cholate residual.

### 3.13. Immunomodulatory Activity of Extract

#### 3.13.1. Cell Culture

RAW264.7 cells were cultivated in a humid atmosphere with 5% CO_2_ in RPMI-1640 complete medium at 310.15 K in a box. The 10% fetal bovine serum (FBS), 100 u/mL penicillin, 1 mmol/L sodium pyruvate, and 100 mg/mL streptomycin were included in this medium.

#### 3.13.2. Cell Viability

The RAW264.7 cell viability was assessed using the MTT assay [45]. Specifically, the RAW264.7 cell suspension (1 × 10^5^ cells/mL) was pipetted into a 96-well plate (100 μL), where it was cultivated for 24 h at 310.15 K in a humid incubator with 5% CO_2_. The cells were treated with 100 μL of different concentrations (12.5, 25, 50, 100, and 200 μg/mL) of the EUL extract, and cultivated for an additional 24 h after discarding the supernatant. A blank control group and a positive control group (lipopolysaccharide, 1 μg/mL) were used at the same time. Then, the supernatant was aspirated. After adding 10 μL of 5 mg/mL MTT solution, the mixture was incubated for 4 h at 310.15 K. Finally, the medium was properly aspirated before each well was given 100 μL of DMSO, shaken for 10 min, and the optical density (OD) was determined at 570 nm using a microplate reader. The calculation formula is as follows:

Cell viability% = A_sample_/A_blank_ × 100% (4)

### 3.14. Anti-Inflammatory Activity of EUL Extract

To further investigate the anti-inflammatory activity of EUL extract, the RAW264.7 cell in logarithmic growth period was counted and their cell density was adjusted. Then, 1 mL of cell suspension was added to a 24-well cell culture plate with 2 × 10^5^ cell per well, and incubated for 24 h at 310.15 K with 5% CO_2_. To induce macrophage polarization, 1.0 μg/mL of lipopolysaccharide (LPS) was added for 8 h, followed by 24 h of EUL extract solution (12.5, 25, 50, 100, and 200 μg/mL). After centrifuging, the cell supernatant was collected to detect the amounts of nitric oxide (NO), interleukin 6 (IL-6), and tumor necrosis factor-α (TNF-α) by a NO kit and a mouse cytokine (IL-6, TNF-α) ELISA kit.

## 4. Conclusions

Ten types of eco-friendly, low-cost, and environmentally favorable DESs were created to extract iridoids, chlorogenic acid, and flavonoids from *Eucommia ulmoides* seed-draff, and the ChAce DES demonstrated the greatest extraction yield of all of them. The extraction yield of CGA, GPA, AU, GP, RU, and IQU were 42.8, 137.2, 156.7, 5.4, 13.5, and 12.8 mg/g, respectively under the optimized parameters (HBD-HBA molar ratio of 1.96, liquid-solid ratio of 28.89 mL/g, water percentage of 38.44%, temperature of 317.36 K, and time of 55.59 min). In addition, the HPD950 resin was the most efficient macroporous resin with excellent reuse stability for recovering desired compounds, as well as a recovery yield of 80.8%, 82.9%, 81.2%, 80.6%, 85.3%, and 79.8% for AU, GPA, GP, CGA, RU, and IQU, respectively. Moreover, the EUL extract had significant hypoglycemic and hypolipidemic activity. At the same time, EUL extract was able to boost macrophage proliferation and regulate immunological activity, and the immunomodulatory activity assay showed that EUL extract exerted notable anti-inflammatory effects. The correlation analysis demonstrated that biological activity was strongly associated with six active compounds. We believe that our work may open up new avenues for the eventual development of an innovative, sustainable, and highly effective method for the specific extraction as well as the utilization of natural products from waste biomass.

## Figures and Tables

**Table 1:** The physical properties of the DESs and sample before and after extraction.

DESs	PH	Viscosity (mPa·s)	S_BET_ (m^2^/g)		V_pore_ (cm^3^/g)	
			Before Extraction	After Extraction	Before Extraction	After Extraction
ChUre	7.26	16.5	2.68	18.55	1.15	0.21
ChAce	2.85	15.6	2.68	25.50	1.15	0.26
ChPro	2.26	13.8	2.68	21.04	1.15	0.24
ChCit	0.48	27.6	2.68	12.93	1.15	0.17
ChOxa	0.11	50.8	2.68	11.81	1.15	0.12
ChMal	0.22	560.1	2.68	14.08	1.15	0.18
ChLac	0.85	26.9	2.68	11.11	1.15	0.23
ChLev	1.09	103.0	2.68	18.48	1.15	0.24
ChGly	6.75	876.2	2.68	23.12	1.15	0.22
ChEG	6.89	378.1	2.68	20.92	1.15	0.20

**Table 2:** The textural parameters of macroporous resin.

Resins	Surface Area (m^2^/g)	Pore Size (Å)	Polarity	Composition
ADS-8	450	130	Non-polar	Polystyrene
HP20	600	290	Non-polar	SDVB
XAD-1	900	95	Non-polar	Polystyrene
H103	1000	95	Non-polar	SDVB
NKA-2	200	150	Middle-polar	Polystyrene
XAD-1180	600	150	Middle-polar	SDVB
LS-A40	700	150	Middle-polar	SDVB
EXA-45	1000	35	Middle-polar	SDVB
ADS-11	210	280	Polar	Sulfonic group
S8	550	100	Polar	Polystyrene
LS-305	1000	55	Polar	SDVB
HPD950	1350	100	Polar	SDVB

## Data Availability

Data are contained within the article and Supplementary Materials.

## Supplementary Materials

The following are available online at https://www.mdpi.com/article/10.3390/molecules29030737/s1, Figure S1: The DTA curves of 10 kinds of DESs, Figure S2: the DSC curves of 10 kinds of DESs; Figure S3: the DTA curves of ChAce with HBA-HBD molar ratio of 1:1, 1:2, 1:3, and 1:4; Figure S4: the DSC curves of ChAce with HBA-HBD molar ratio of 1:1, 1:2, 1:3, and 1:4; Figure S5–S10: response surface for the interactions of independent variables on CGA, GPA, AU, GP, RU, and IQU amount; Figure S11: HPLC chromatograms of standard components and EUL extract; Table S1: ANOVA analysis results of the quadratic model; Table S2: model equations with coded factors for the amounts of 6 target components; Table S3: repeatability of the extraction; Table S4: design factor levels and codes for extraction; Table S5: the specific factor level design for iridoids, chlorogenic acid, and flavonoids extraction.
