# Supplementary material for: Combination of a Deep Eutectic Solvent and Macroporous Resin for Green Recovery of Iridoids, Chlorogenic Acid, and Flavonoids from Eucommia ulmoides Leaves
Source: Molecules. 2024 Feb 5;29(3):737. doi: 10.3390/molecules29030737 (PMC10856201; doi:10.3390/molecules29030737)
Supplement: Supplementary file 1 [file molecules-29-00737-s001.zip › molecules-2840878-supplementary.pdf]

Supplementary Materials

Combination of a Deep Eutectic Solvent and Macroporous Resin for Green Recovery of Iridoids, Chlorogenic Acid, and Flavonoids from *Eucommia ulmoides* Leaves

Yunhui Liao [1,2], Feng Chen [1], Haishan Tang [1,3], Wubliker Dessie [1,2] and Zuodong Qin [1,2,*]

[1] College of Chemistry and Bioengineering, Hunan University of Science and Engineering, Yongzhou 425199, China; liaoyunhui@huse.edu.cn (Y.L.); chenfeng1157354@163.com (F.C.); thshappy@163.com (H.T.); dwubliker@yahoo.com (W.D.)

[2] Hunan Engineering Technology Research Center for Comprehensive Development and Utilization of
Biomass Resources, College of Chemistry and Bioengineering, Hunan University of Science and Engineering, Yongzhou 425199, China

[3] Hunan Provincial Key Laboratory for Comprehensive Utilization of Dominant Plant Resources in Southern Hunan, Yongzhou 425199, China

* Correspondence: dong6758068@163.com

1. Thermal properties of DESs

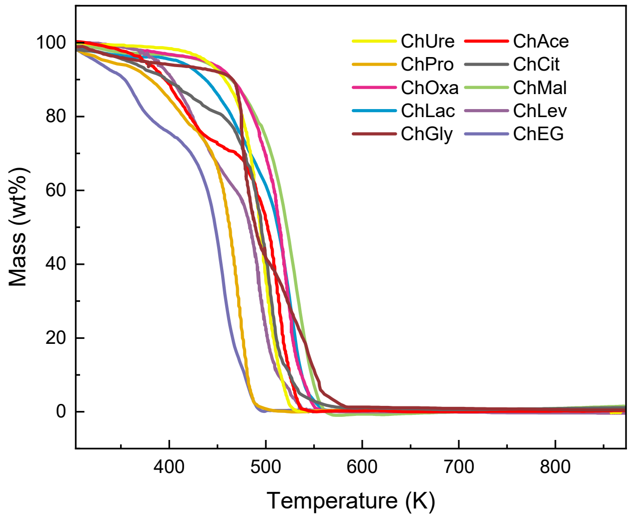

Figure S1. The DTA curves of 10 kinds of DESs

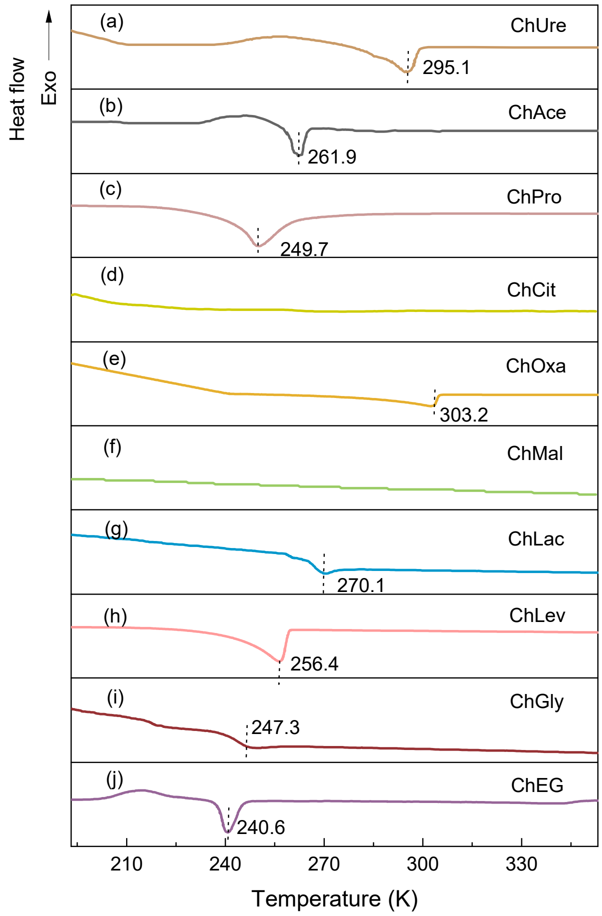

Figure S2. The DSC curves of 10 kinds of DESs

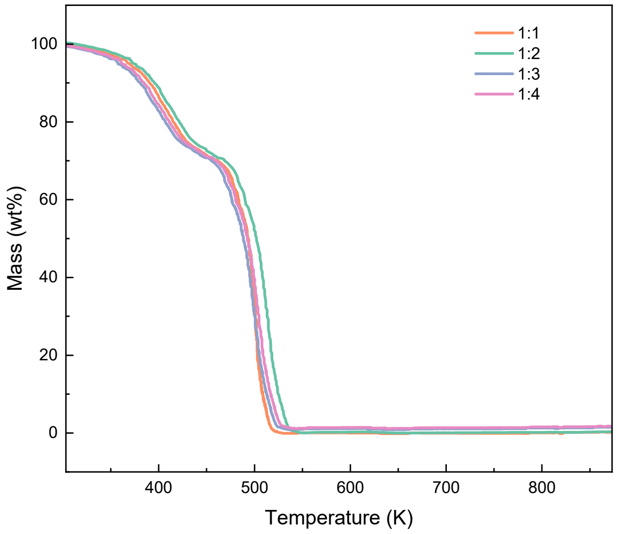

Figure S3. The DTA curves of ChAce with HBA-HBD molar ratio of 1:1, 1:2, 1:3, and 1:4.

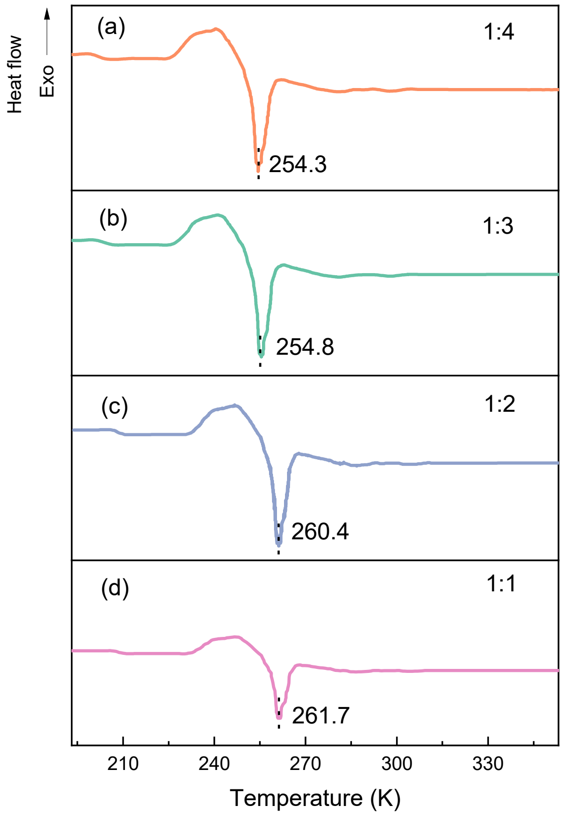

Figure S4. The DSC curves of ChAce with HBA-HBD molar ratio of 1:1, 1:2, 1:3, and 1:4.

2. Response surface methodology (RSM)

Table S1. ANOVA analysis results of the quadratic model.

Source	CGA		GPA		AU		GP		RU		IQU	
	F-value	P-value	F-value	P-value	F-value	P-value	F-value	P-value	F-value	P-value	F-value	P-value
Model	320.26	< 0.0001	382.08	< 0.0001	512.64	< 0.0001	55.44	< 0.0001	86.09	< 0.0001	371.94	< 0.0001
A	13.29	0.0012	79.84	< 0.0001	173.98	< 0.0001	22.68	< 0.0001	80.11	< 0.0001	249.93	< 0.0001
B	28.02	< 0.0001	273.18	< 0.0001	100.78	< 0.0001	15.93	0.0005	43.35	< 0.0001	27.77	< 0.0001
C	22.07	< 0.0001	97.70	< 0.0001	81.70	< 0.0001	5.27	0.0304	1.79	0.1931	37.37	< 0.0001
D	22.07	< 0.0001	2.70	0.1128	19.66	0.0002	31.38	< 0.0001	33.19	< 0.0001	111.08	< 0.0001
E	161.93	< 0.0001	457.51	< 0.0001	375.57	< 0.0001	38.89	< 0.0001	85.73	< 0.0001	115.57	< 0.0001
AB	35.51	< 0.0001	0.2143	0.6474	3.85	0.0609	4.51	0.0437	4.23	0.0502	34.83	< 0.0001
AC	0.0197	0.8896	23.63	< 0.0001	123.95	< 0.0001	7.33	0.0121	28.62	< 0.0001	45.50	< 0.0001
AD	104.78	< 0.0001	91.85	< 0.0001	20.51	0.0001	1.44	0.2417	4.23	0.0502	149.47	< 0.0001
AE	14.87	0.0007	60.50	< 0.0001	64.01	< 0.0001	9.09	0.0058	1.06	0.3134	34.83	< 0.0001
BC	17.11	0.0003	2.78	0.1081	133.37	< 0.0001	0.8558	0.3637	1.06	0.3134	129.56	< 0.0001
BD	21.41	< 0.0001	21.01	0.0001	4.15	0.0523	0.0188	0.8921	5.12	0.0325	21.50	< 0.0001
BE	3.07	0.0919	51.50	< 0.0001	0.0057	0.9404	0.0188	0.8921	2.71	0.1123	8.71	0.0068
CD	90.92	< 0.0001	214.04	< 0.0001	60.44	< 0.0001	41.49	< 0.0001	38.10	< 0.0001	344.08	< 0.0001
CE	240.08	< 0.0001	67.91	< 0.0001	33.78	< 0.0001	10.37	0.0035	6.10	0.0207	57.58	< 0.0001
DE	8.67	0.0069	2.06	0.1636	0.2407	0.6280	0.3804	0.5430	2.07	0.1622	0.7109	0.4071
A^2	2812.31	< 0.0001	5243.96	< 0.0001	8606.32	< 0.0001	771.79	< 0.0001	1330.15	< 0.0001	4706.17	< 0.0001
B^2	1011.33	< 0.0001	1099.69	< 0.0001	1178.56	< 0.0001	221.28	< 0.0001	240.26	< 0.0001	488.68	< 0.0001
C^2	1035.63	< 0.0001	322.46	< 0.0001	120.40	< 0.0001	101.35	< 0.0001	83.13	< 0.0001	202.17	< 0.0001
D^2	2596.93	< 0.0001	1609.15	< 0.0001	908.97	< 0.0001	287.89	< 0.0001	100.59	< 0.0001	1963.91	< 0.0001
E^2	3217.71	< 0.0001	1715.08	< 0.0001	882.28	< 0.0001	211.84	< 0.0001	204.05	< 0.0001	1909.10	< 0.0001
Lack of Fit	0.2562	0.9876	0.1061	0.9999	2.30	0.1805	0.3717	0.9487	0.9033	0.6122	2.39	0.1699
R^2	0.9930		0.9941		0.9956		0.9603		0.9742		0.9940	
Adj-R^2	0.9894		0.9928		0.9909		0.9345		0.9507		0.9874	

Table S2. Model equations with coded factors for the amounts of 6 target components.

Model	Equation
CGA amount (mg/g)	$= 43.1-0.65A+0.944B-0.838C-0.838D-2.269E-2.125AB-0.050AC-3.65AD+1.375AE-1.475BC+1.65BD+0.625BE+3.4CD-5.525CE+1.05DE-12.802A^2-7.677B^2-7.769C^2-12.302D^2-13.694E^2$
GPA amount (mg/g)	$=136.133-2.413A-4.463B-2.669C-0.444D-5.775E-0.250AB-2.625AC-5.175AD+4.2AE+0.9BC+2.475BD-3.875BE+7.9CD-4.45CE+0.775DE-26.473A^2-12.123B^2-6.565C^2-14.665D^2-15.140E^2$

AU amount (mg/g)	$=155.35-4.369A-3.325B-2.994C-1.469D-6.419E-1.3AB-7.375AC-3AD+5.3AE+7.65BC+1.35BD+0.050BE+5.15CD-3.85CE-0.325DE-41.604A^2-15.396B^2-4.921C^2-13.521D^2-13.321E^2$
GP amount (mg/g)	$=5.448-0.174A-0.146B-0.084C-0.204D-0.228E-0.155AB-0.198AC-0.088AD+0.22AE-0.068BC+0.01BD+0.010BE+0.47CD-0.235CE+0.045DE-1.372A^2-0.735B^2-0.497C^2-0.838D^2-0.719E^2$
RU amount (mg/g)	$=13.4-0.544A-0.4B-0.081C-0.35D-0.563E-0.25AB-0.65AC+0.25AD+0.125AE+0.125BC-0.275BD-0.2BE+0.75CD-0.3CE+0.175DE-3A^2-1.275B^2-0.75C^2-0.825D^2-1.175E^2$
IQU amount (mg/g)	$=12.833-0.469A-0.156B-0.181C-0.313D-0.319E-0.35AB-0.4AC-0.725AD+0.35AE+0.675BC-0.275BD-0.175BE+1.1CD-0.45CE+0.05DE-2.754A^2-0.888B^2-0.571C^2-1.779D^2-1.754E^2$

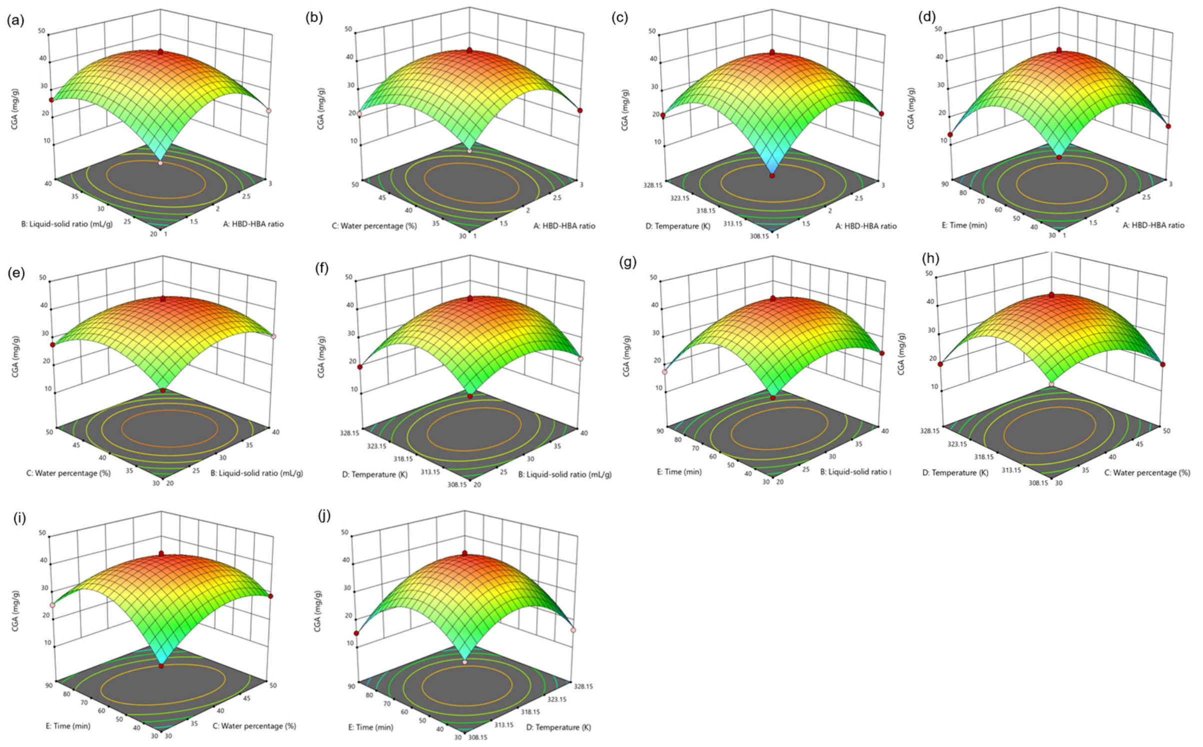

Figure S5. Response surface for the interactions of independent variables on CGA amount (a: HBD-HBA ratio and liquid-solid ratio; b: HBD-HBA ratio and water percentage; c: HBD-HBA ratio and temperature; d: HBD-HBA ratio and time; e: Liquid-solid ratio and water percentage; f: Liquid-solid ratio and temperature; g: Liquid-solid ratio and time; h: Water percentage and temperature; i: Water percentage and time; j: Temperature and time).

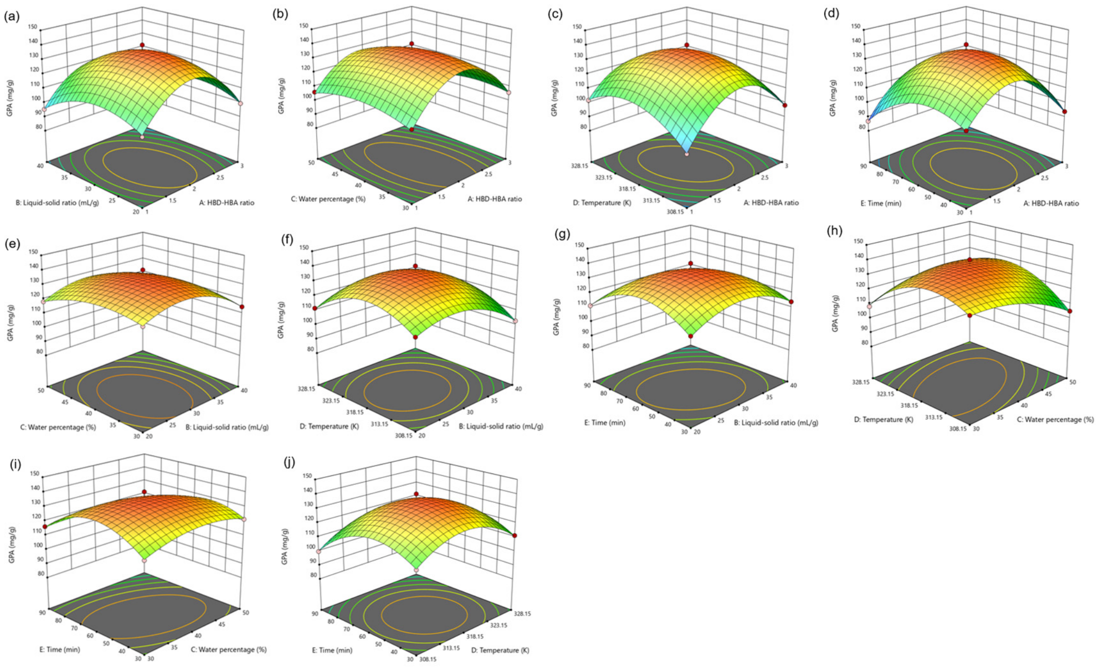

Figure S6. Response surface for the interactions of independent variables on GPA amount (a: HBD-HBA ratio and liquid-solid ratio; b: HBD-HBA ratio and water percentage; c: HBD-HBA ratio and temperature; d: HBD-HBA ratio and time; e: Liquid-solid ratio and water percentage; f: Liquid-solid ratio and temperature; g: Liquid-solid ratio and time; h: Water percentage and temperature; i: Water percentage and time; j: Temperature and time).

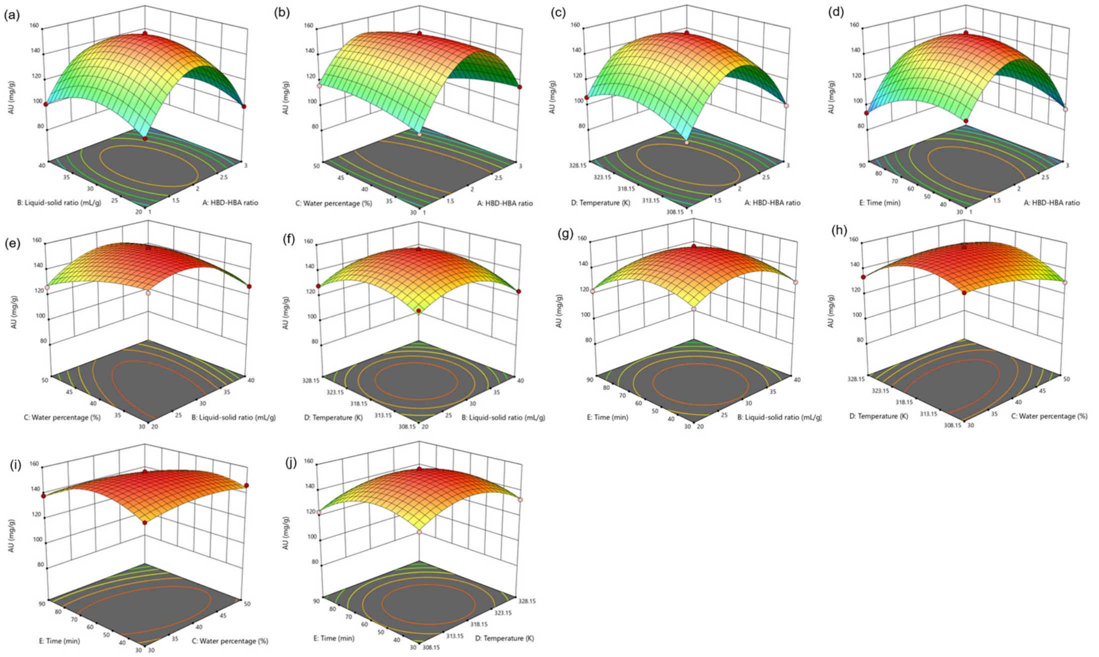

Figure S7. Response surface for the interactions of independent variables on AU amount (a: HBD-HBA ratio and liquid-solid ratio; b: HBD-HBA ratio and water percentage; c: HBD-HBA ratio and temperature; d: HBD-HBA ratio and time; e: Liquid-solid ratio and water percentage; f: Liquid-solid ratio and temperature; g: Liquid-solid ratio and time; h: Water percentage and temperature; i: Water percentage and time; j: Temperature and time).

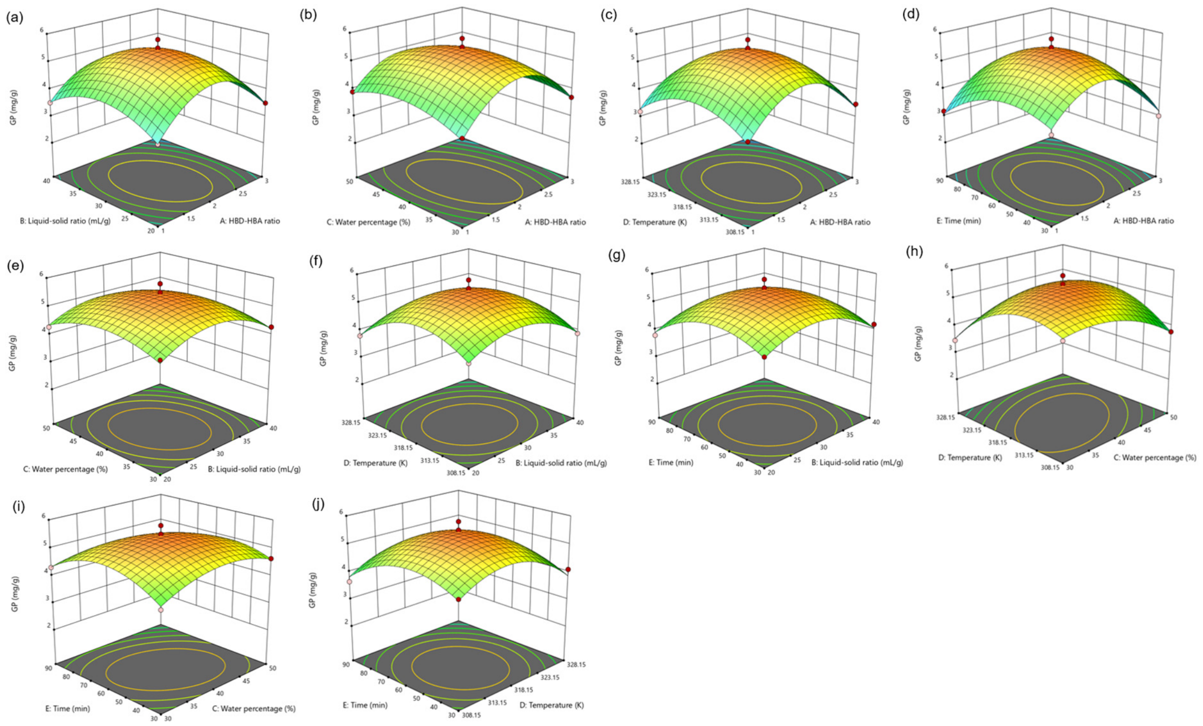

Figure S8. Response surface for the interactions of independent variables on GP amount (a: HBD-HBA ratio and liquid-solid ratio; b: HBD-HBA ratio and water percentage; c: HBD-HBA ratio and temperature; d: HBD-HBA ratio and time; e: Liquid-solid ratio and water percentage; f: Liquid-solid ratio and temperature; g: Liquid-solid ratio and time; h: Water percentage and temperature; i: Water percentage and time; j: Temperature and time).

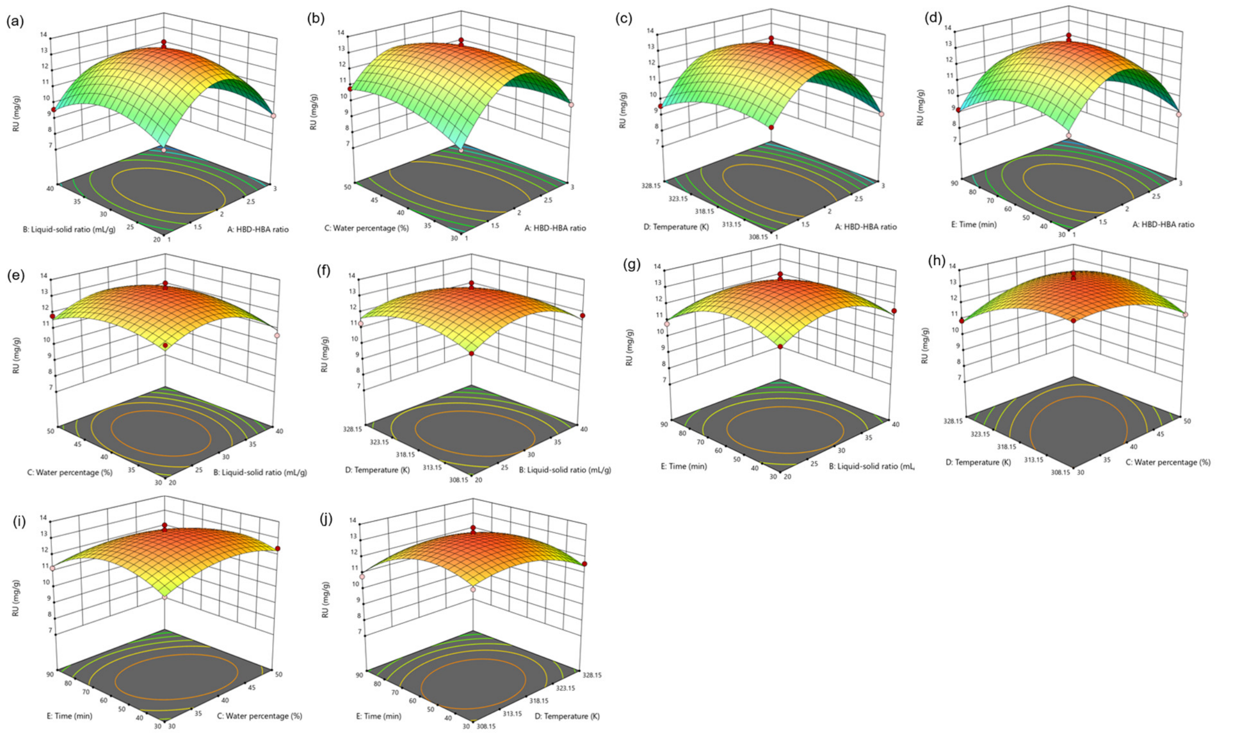

Figure S9. Response surface for the interactions of independent variables on RU amount (a: HBD-HBA ratio and liquid-solid ratio; b: HBD-HBA ratio and water percentage; c: HBD-HBA ratio and temperature; d: HBD-HBA ratio and time; e: Liquid-solid ratio and water percentage; f: Liquid-solid ratio and temperature; g: Liquid-solid ratio and time; h: Water percentage and temperature; i: Water percentage and time; j: Temperature and time).

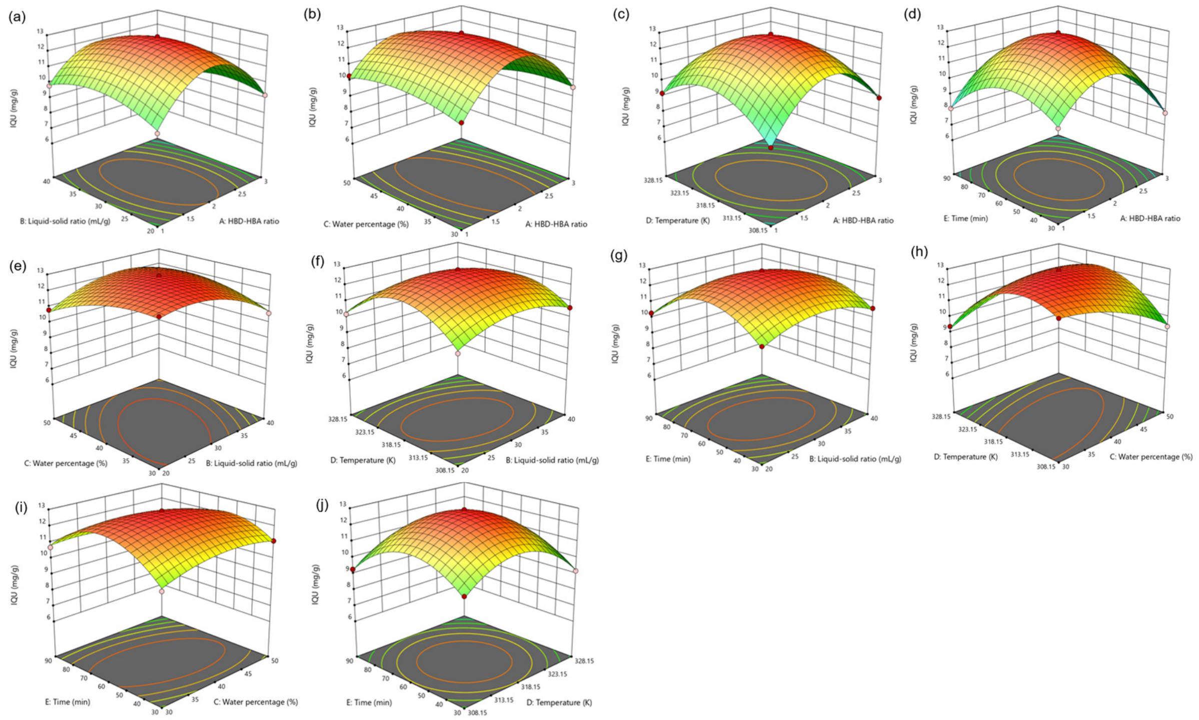

Figure S10. Response surface for the interactions of independent variables on IQU amount (a: HBD-HBA ratio and liquid-solid ratio; b: HBD-HBA ratio and water percentage; c: HBD-HBA ratio and temperature; d: HBD-HBA ratio and time; e: Liquid-solid ratio and water percentage; f: Liquid-solid ratio and temperature; g: Liquid-solid ratio and time; h: Water percentage and temperature; i: Water percentage and time; j: Temperature and time).

3. Repeatability test

Table S3. Repeatability of the extraction*

Amount (mg/g)	Entry							Average (mg/g)	RSD (%) (n=7)	RSD (%) average
	1	2	3	4	5	6	7			
CGA	42.1	42.5	42.7	42.4	42.6	42.5	42.4	42.4	0.44	0.73
GPA	137.1	137.1	136.2	136.1	136.8	136.8	136.8	136.7	0.40	
AU	155.3	156.3	155.3	153.6	154.9	155.3	153.6	154.9	0.64	
GP	5.2	5.2	5.3	5.3	5.2	5.1	5.2	5.2	1.32	
RU	13.1	13.4	13.2	13.4	13.4	13.3	13.1	13.2	1.04	
IQU	12.6	12.5	12.6	12.6	12.6	12.7	12.7	12.6	0.55	

*Extraction conditions: HBD-HBA ratio of 1.96, liquid-solid ratio of 28.89 mL/g, water percentage of 38.44%, temperature of 317.15 K, and time of 55.60 min.

4. HPLC chromatograms of standard components and EUL extract

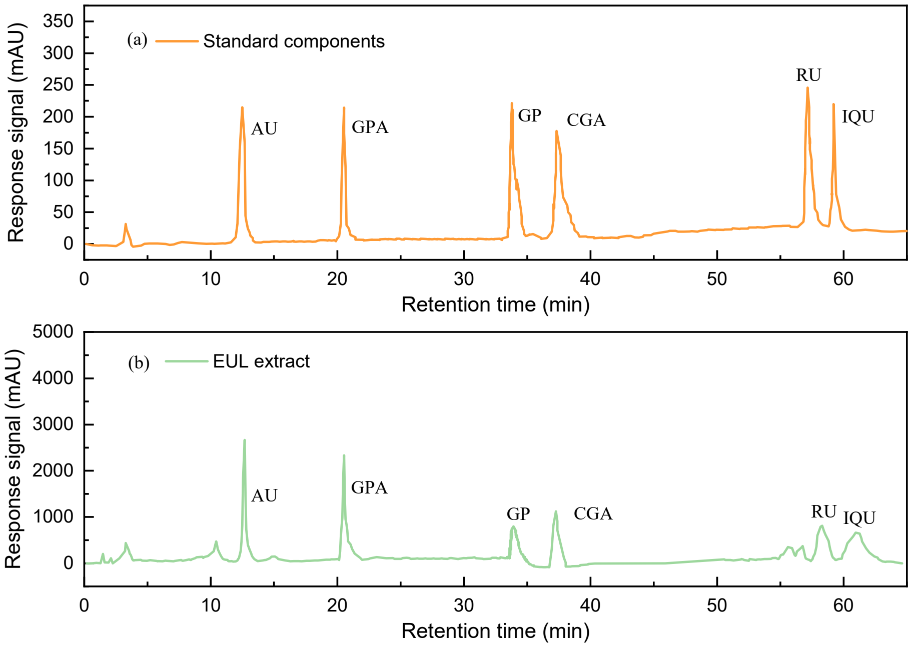

Figure S11. HPLC chromatograms of (a) standard components and (b) EUL extract.
Detection concentration: 1.0 mg/mL (standard components) and 10 mg/mL (EUL extract). Detection wavelength: 206 nm (AU), 238 nm (GPA and GP), 254 nm (RU and IQU), and 320 nm (CGA).

5. Response surface optimization design of experiments

Five factors of DESs composition molar ratio, liquid-solid ratio (mL/g), water percentage (%), extraction temperature (K), and time (min) were employed as experimental reference factors, while extraction amount was served as the response value. The factor level coding table of Box-Behnken experimental design is shown in Table S4. The specific factor level design is shown in Table S5.

Table S4. Design factor levels and codes for extraction

Name	Units	Type	Low	High
HBD-HBA ratio		Factor	1	3
Liquid-solid ratio	mL/g	Factor	20	40
Water percentage	%	Factor	30	50
Temperature	K	Factor	308.15	328.15
Time	min	Factor	30	90
CGA amount	mg/g	Response		
GPA amount	mg/g	Response		
AU amount	mg/g	Response		
GP amount	mg/g	Response		

RU amount	mg/g	Response
IQU amount	mg/g	Response
Total	mg/g	Response

Table S5. The specific factor level design for iridoids, chlorogenic acid, and flavonoids extraction

Std	Run	A: HBD-HBA ratio	B: Liquid-solid ratio (mL/g)	C: Water percentage (%)	D: Temperature (k)	E: Time (min)	Amount (mg/g)						
							CGA	GPA	AU	GP	RU	IQU	Total
28	1	3	30	40	328.15	60	13.2	87.2	91.8	2.7	8.9	6.8	210.6
9	2	2	20	40	318.15	30	23.8	115.8	135.6	4.4	11.8	10.6	302
6	3	2	30	50	308.15	60	19.7	105.1	129.6	3.8	11.3	9.4	278.9
20	4	2	30	40	328.15	90	15.2	101.4	120.3	3.5	10.8	8.6	259.8
37	5	2	20	40	308.15	60	24.8	117.1	135.6	4.2	11.8	10.2	303.7
13	6	1	30	30	318.15	60	23.8	106.4	108.3	3.7	9.6	9.9	261.7
23	7	2	20	50	318.15	60	27.8	118.3	126.3	4.3	11.8	10.8	299.3
36	8	3	30	40	318.15	90	14.6	90.2	94.2	3.3	8.4	7.9	218.6
22	9	2	40	30	318.15	60	30.8	114.8	127.1	4.3	10.6	10.6	298.2
38	10	2	40	40	308.15	60	22.8	102.8	123.9	3.9	11.8	10.6	275.8
46	11	2	30	40	318.15	60	44.3	140.2	155.2	5.5	13.2	12.8	371.2
35	12	1	30	40	318.15	90	13.8	86.9	93.8	3.2	9.2	8.1	215
25	13	1	30	40	308.15	60	15.9	92.3	102.2	3.6	10.8	8.4	233.2
45	14	2	30	40	318.15	60	43.6	135.2	156.7	5.8	13.5	12.9	367.7
2	15	3	20	40	318.15	60	22.8	99.6	99.3	3.5	9.2	9.2	243.6
34	16	3	30	40	318.15	30	16.9	93.8	96.8	3	8.9	7.8	227.2
3	17	1	40	40	318.15	60	26.8	95.4	101.1	3.5	9.6	9.8	246.2
8	18	2	30	50	328.15	60	25.3	118.7	137.2	4.3	12.1	11.1	308.7
41	19	2	30	40	318.15	60	43.8	135.4	154.6	5.4	13.3	12.8	365.3
40	20	2	40	40	328.15	60	24.4	107.2	121.8	3.5	10.2	9.5	276.6
17	21	2	30	40	308.15	30	20.8	112.9	134.8	4.4	12.3	10.1	295.3
10	22	2	40	40	318.15	30	24.5	114.1	129.3	4.2	11.6	10.6	294.3
31	23	2	30	30	318.15	90	25.6	116.3	138.1	4.3	11.2	10.7	306.2
12	24	2	40	40	318.15	90	20.8	94.1	116.2	3.6	9.8	9.6	254.1
44	25	2	30	40	318.15	60	41.6	135.9	155.8	5.5	13.5	12.7	365
21	26	2	20	30	318.15	60	26.5	125.2	147.2	4.5	12.3	12.5	328.2

30	27	2	30	50	318.15	30	28.9	121.4	146.3	4.6	12.4	11.1	324.7
11	28	2	20	40	318.15	90	17.6	111.3	122.3	3.8	10.8	10.3	276.1
29	29	2	30	30	318.15	30	19.3	117.9	143.2	4.2	11.8	10.4	306.8
4	30	3	40	40	318.15	60	21.5	90.8	90.1	2.9	8.1	8.3	221.7
24	31	2	40	50	318.15	60	26.2	111.5	136.8	3.8	10.6	11.6	300.5
43	32	2	30	40	318.15	60	41.8	134.8	154.1	5.2	13.1	12.9	361.9
14	33	3	30	30	318.15	60	22.8	105.9	115.2	3.7	9.8	9.6	267
16	34	3	30	50	318.15	60	20.4	95.2	93.6	3.1	8.4	8.4	229.1
27	35	1	30	40	328.15	60	21.6	101.9	106.3	3.2	9.6	9.2	251.8
26	36	3	30	40	308.15	60	22.1	98.3	99.7	3.5	9.1	8.9	241.6
19	37	2	30	40	308.15	90	15.3	99.9	123.3	3.7	10.8	9.3	262.3
1	38	1	20	40	318.15	60	19.6	103.2	105.1	3.5	9.7	9.3	250.4
33	39	1	30	40	318.15	30	21.6	107.3	117.6	3.8	10.2	9.4	269.9
42	40	2	30	40	318.15	60	43.5	135.3	155.7	5.3	13.8	12.9	366.5
15	41	1	30	50	318.15	60	21.6	106.2	116.2	3.9	10.8	10.3	269
32	42	2	30	50	318.15	90	13.1	102	125.8	3.8	10.6	9.6	264.9
7	43	2	30	30	328.15	60	19.8	108.3	133.8	3.5	10.9	9.4	285.7
18	44	2	30	40	328.15	30	16.5	111.3	133.1	4.1	11.6	9.2	285.8
39	45	2	20	40	328.15	60	19.8	111.6	128.1	3.8	11.3	10.2	284.8
5	46	2	30	30	308.15	60	27.8	126.3	146.8	4.8	13.1	12.1	330.9
